# Humans as Source of *Mycobacterium tuberculosis* Infection in Cattle, Spain

**DOI:** 10.3201/eid1712.101476

**Published:** 2011-12

**Authors:** Beatriz Romero, Sabrina Rodríguez, Javier Bezos, Rosa Díaz, M. Francisca Copano, Isabel Merediz, Olga Mínguez, Sergio Marqués, Juan J. Palacios, Darío García de Viedma, José Luis Sáez, Ana Mateos, Alicia Aranaz, Lucas Domínguez, Lucía de Juan

**Affiliations:** Universidad Complutense, Madrid, Spain (B. Romero, S. Rodríguez, J. Bezos, A. Mateos, A. Aranaz, L. Domínguez, L. de Juan);; Consejería de Medio Ambiente, Vivienda y Ordenación del Territorio, Madrid (R. Díaz);; Laboratorio de Sanidad Animal de Jove, Gijón, Spain (M.F. Copano, I. Merediz);; Consejería de Agricultura y Ganadería, Valladolid, Spain (O. Mínguez, S. Marqués);; Hospital Universitario Central de Asturias, Oviedo, Spain (J.J. Palacios);; Hospital General Universitario Gregorio Marañón, Madrid, and Centro de Investigación Biomédica en Red Enfermedades Respiratorias Enfermedades Respiratorias, Madrid (D. García de Viedma);; Ministerio de Medio Ambiente, y Medio Rural y Marino, Madrid (J.L. Sáez)

**Keywords:** Mycobacterium tuberculosis, human, cattle, epidemiology, bacteria, drug resistance, zoonoses, tuberculosis and other mycobacteria

**To the Editor:** *Mycobacterium tuberculosis* is the main causative agent of tuberculosis in humans. However, little attention has been paid to its transmission from humans to animals. We report *M. tuberculosis* infections in 3 cattle farms in Spain. The epidemiologic investigation traced humans as the source of infection, with 1 of the strains showing multidrug resistance.

Recent studies have reported isolation of *M. tuberculosis* in cattle with prevalences of 4.7%–30.8% in African and Asian countries (*1–**3*). In cattle, this infection occurs in countries with the highest incidence of human tuberculosis in the world. In Europe, only 14 cases of *M. tuberculosis* infection have been described in 3 eastern countries since implementation of eradication programs (*4**,**5*). The only reported cases of *M. tuberculosis* in cattle in western Europe were described in Great Britain and date back to the 1950s (*6*).

During 2007–2009, three cases of tuberculosis caused by *M. tuberculosis* were detected in 3 unrelated cattle farms, 2 of them free of tuberculosis (farms 1 and 2). As part of the surveillance system of bovine tuberculosis, a pool of tissue samples from each cow (respiratory lymph nodes and lung) were homogenized with sterile distilled water, and culture was carried out by the BACTEC mycobacteria growth indicator tube 960 system (Beckton Dickinson, Madrid, Spain). Members of the *M. tuberculosis* complex were identified and genotyped by direct variable repeat spacer olignucleotide typing and mycobacterial interspersed repetitive unit–variable number tandem repeat (MIRU-VNTR) typing (*7*).

The 3 *M. tuberculosis*–infected animals were <9 months of age (Table). As described (*6*), the possibility of infection in young animals could be more probable than infection in older cows.

**Table:** Relevant information about *Mycobacterium tuberculosis* infection in 3 cattle farms in Spain*

Variable	Farm 1	Farm 2	Farm 3
Cattle herd			
No. animals	6	54	>200
Production	Beef	Beef	Dairy
Previous status	TB free	TB-free	Non–TB free
No. reactors	1	31	12
*M. tuberculosis* infection in cattle			
Year of isolation	2007	2008	2009
Age, mo	9	4	3
IDTB/interferon-γ	Pos/not determined	Neg/neg	Pos/not determined
TB-compatible lesion	No	No	No
Spoligotyping profile	SIT2537	SIT1564	SIT58
MIRU-VNTR profile†	253533233433236252211423	3′52334232455457251213423	254343243232325262213423
Co-infection with other mycobacteria	Yes (*M. avium* subsp. *hominissuis*)‡	Yes (*M. bovis*)	No
*M. tuberculosis* infection in human			
Active tuberculosis	Yes	Yes	Yes
Spoligotyping profile	SIT2537	Not available	SIT58
MIRU-VNTR profile†	253533233433236252211423	3′52334232455457251213423	254343243232325262213423
Origin	Spain	Eastern Europe	Spain

*TB, tuberculosis; IDTB, intradermal tuberculin tested according to the European Council Directive 64/432/EEC; pos, positive; neg, negative; MIRU-VNTR, mycobacterial interspersed repetitive unit–variable number tandem repeat. †MIRU-VNTR profile on the basis of the 24 MIRU-VNTR loci (*7*). ‡Co-infection in the same animal.

*M. tuberculosis*–infected animals from farms 1 and 3 were detected by the intradermal tuberculin test (Table). The animal without immunologic response (farm 2) was detected because an *M. bovis* infection was confirmed in the herd, and all animals were slaughtered. Confirmation of infection by culture without immunologic response is rare, although the high sensitivity of the mycobacteria growth indicator tube system could detect a low bacterial load in the initial stages of infection. Recent implementation of liquid systems in animal health laboratories has enabled detection of *M. tuberculosis* when it is compared with results using only conventional methods. Moreover, no tuberculosis-compatible lesions were observed in the 3 animals, similar to previous studies (*6*). On the basis of these facts, *M. tuberculosis* transmission was not detected among cattle in the following intradermal tuberculin tests.

Co-infection with other mycobacteria (*M. avium* subsp. *hominissuis*) was found in the same animal from farm 1 (Table). This co-infection suggested the immunocompromised status of the animal and hence a high susceptibility to *M. tuberculosis* infection. Moreover, *M. bovis* was isolated from 52% (16/31) of all animals from farm 2 that showed a positive reaction to the intradermal tuberculin skin test, making remarkable the absence of co-infection with *M. bovis* in the *M. tuberculosis*–infected animal. Therefore, the lack of *M. tuberculosis* transmission within this herd contrasts with the *M. bovis* dissemination.

The veterinary services reported these findings to the National Public Health System, and an epidemiologic investigation was conducted on the cattle farms to determine the source of infection. In all cases, staff of the farms had active tuberculosis (Table). Three different strains were characterized: SIT2537 (octal code 777617777720771), 253533233433236252211423 (farm 1); SIT1564, 3′52334232455457251213423 (farm 2); and SIT58, 254343243232325262213423 (farm 3) (Table). The MIRU-VNTR pattern and spoligotype are shared by Spanish human and cattle isolates from farm 1; SIT2537 is an uncommon profile that has been detected in Brazil and Spain (according to the SITVIT2 database). The human strain showed multidrug resistance to isoniazid, rifampin, and ethionamide. In cattle and human isolates, genes associated with isoniazid and rifampin resistance were studied (*8*) and *rpoB* analysis confirmed rifampin resistance (Ser531Leu). In farm 2, the origin of the farm worker was eastern Europe and the cattle isolate showed an SIT1564 profile, which is found only in 6 human isolates in the SpolDB4 database, all from Poland, Bulgaria, and Russia. On farm 3, human and cattle isolates from Spain shared identical spoligotype and MIRU-VNTR patterns. The profile SIT58 is frequent in Spain (*9*) and other countries with historical links to Spain, mainly the south American countries (79/114 according to SpolDB4).

A well-designed program for eradicating bovine tuberculosis helps to detect *M. tuberculosis* infection by immune response or by bacteriologic culture. The use of liquid systems and results of epidemiologic studies (Spanish Database of Animal Mycobacteriosis, mycoDB.es) (S. Rodríguez, unpub. data) are recommended for prompt confirmation of the *M. tuberculosis* complex infection and for enhancing the sensitivity of culture. In addition, the Spanish Ministry of Environment, Rural and Marine Affairs has reinforced the need to improve cooperation between human and animal health systems to minimize the risk for *M. tuberculosis* complex transmission from animals to humans or vice versa and to control infection in all susceptible animal species (*10*).
